# The forms and adverse effects of insecurities among internally displaced children in Ethiopia

**DOI:** 10.1186/s12889-023-15109-9

**Published:** 2023-01-30

**Authors:** Janet Kemei, Bukola (Oladunni) Salami, Matiwos Soboka, Hayat Imam Mohamed Gommaa, Philomina Okeke-Ihejirika, Tina Lavin

**Affiliations:** 1 Faculty of Nursing, University of Alberta, T6G 1C9 Edmonton, Canada; 2 Department of Psychiatry, Faculty of Medicine, Jimma University, Jimma, Ethiopia; 3 Faculty of Allied Health Sciences Nursing Sciences Department, Ahmadu Bello University, Zaria, Nigeria; 4 Department of Women’s, University of Alberta, Edmonton, Canada; 5 School of Global and Population Health, University of Western Australia, Perth, Australia

**Keywords:** African child, Internally displaced children, Insecurities, Social determinants of health

## Abstract

**Background:**

Ethiopia has seen an increase in the number of internally displaced persons (IDPs) due to conflict and violence related to border-based disputes and climate change. This study examines the insecurities experienced by IDPs in the Burayu camp and how they navigate and challenge them. Violence and insecurity have daunted Ethiopian regions for decades, violated children’s rights, and impeded the achievement of the United Nation’s sustainable development goals related to children, such as good healthcare and mental health, quality education, clean water, and sanitation. The deteriorating security concerns in Ethiopia could also expose IDP children to poor health outcomes associated with a lack of access to healthcare services.

**Methods:**

This was an exploratory qualitative case study guided by intersectionality theoretical lens to explore the forms of insecurities perceived and experienced by IDPs in Ethiopia. Participants were selected using a purposeful sampling approach. We interviewed 20 children, 20 parents or guardians, and 13 service providers. Interviews were audio recorded and transcribed verbatim in Afan Oromo, then translated into English. We used NVivo 12 qualitative data analysis software to analyze data following Braun & Clarke’s approach to thematic data analysis.

**Results:**

The participants reported that IDP children in Burayu town faced many challenges related to poor socioeconomic conditions that exposed them to several insecurities and negatively affected their well-being. They reported inadequate access to clothing and shelter, clean water, sanitary facilities, food, and adequate healthcare due to financial barriers, lack of drugs, and quality of care. Our data analysis shows that socioeconomic and contextual factors intersect to determine the health and well-being of children in the Ethiopian IDP camp studied. The children experienced insecurities while navigating their daily lives. This is compounded by institutional practices that shape gender relations, income status, and access to healthcare, education, and food. These deficiencies expose children to traumatic events that could decrease future livelihood prospects and lead to compromised mental health, rendering them susceptible to prolonged post-traumatic stress disorder and depression. Results are presented under the following topics: (1) basic needs insecurity, (2) healthcare insecurity, (3) academic insecurity, (4) economic insecurity, (5) food insecurity, and (6) physical and mental health insecurity.

**Conclusion:**

Successful relocation and reintegration of IDPs would help to alleviate both parent and child post-conflict stressors. Managing and following up on economic reintegration efforts is needed in both the short and long term. Such measures will help to achieve goals for specific projects attached to donor support outcomes, consequently enabling social support and conflict resolution management efforts.

## Background

As of June 2021, the United Nations High Commission on Refugees (UNHCR) reported approximately 82.2 million forcibly displaced people globally. [1] Among the forcibly displaced, 48 million are internally displaced persons (IDPs), 42% of which are children under 18 years of age. [1] IDPs are forced to leave their homes or residences for many reasons, including conflict and violence, climatic shocks causing natural disasters, and other violations of rights. [1], [2].

The International Displacement Monitoring Center (IDMC) reports Ethiopia had the highest number of IDPs anywhere in the world, with just over two million in December 2020. [3] Conflict remains the primary cause of displacement in Ethiopia. [4] In 2020, the IDMC recorded 1,692,000 new displacements in Ethiopia due to war and violence related to border-based disputes. [3] Violence and insecurity have affected Ethiopian regions for decades. This violence threatens children’s rights and impedes efforts to achieve sustainable development goals (SDGs) related to children, such as good healthcare and mental health, quality education, clean water, and sanitation [5].

The literature indicates internally displaced children experience poor health outcomes associated with lack of access to healthcare services and poor sanitation, rendering them vulnerable to infectious diseases and disrupted schooling due to constant movement and language barriers, both of which can impact children’s normal development [4, 6-9]. Similarly, conflicts in the Amhara, Oromia, and Tigray regions have increased food insecurities and, when compounded with desert locust invasion and socioeconomic impacts of COVID-19, threaten severe malnutrition as families struggle with access to basic needs [3]. As the number of IDPs continues to grow with Ethiopian conflicts, the United Nations Office for the Coordination of Humanitarian Affairs (UNOCHA) [10] has therefore warned the crisis will negatively affect food production and inflate food prices, creating extreme food consumption gaps. Further, displaced children may succumb to harmful coping mechanisms, such as early marriages, school dropouts, and skipping meals. [10] Issues of malnutrition and poor health outcomes related to food insecurity are common among IDPs in most African counties. Cumber and colleagues observed that IDPs in Cameroon fleeing attacks from the Boko Haram were at risk of food shortages as the food supplied to them was insufficient and the camps have limited space to cultivate food. [11] Migration also makes it difficult for IDPs to navigate the health system and follow through with immunization against preventable communicable diseases. [6] As such, continuous migration and overcrowding have increased the occurrence of preventable infectious diseases such as polio and measles.

The displacement also underpins pre-existing socioeconomic disadvantages against women and children, increases the risk of gender-based violence against women and girls, [12], [13] and increases morbidity and mortality among women and young children. [14] Insecurity Insight [15] reports the sexual violation of women and girls in some IDP camps in Ethiopia, where they have been raped, assaulted, and killed. Similarly, in Uganda, adult female heads of households have reported abuse directed at them, their sisters, and their neighbors in IDP camps. [13] The abuse exposes these women to unintended pregnancies, sexually transmitted infections such as HIV, and chronic mental health issues such as depression. [13] Further, these traumatic events could decrease future livelihood prospects for internally displaced children and compromise mental health, rendering them vulnerable to prolonged post-traumatic stress disorder (PTSD) and depression. [16], [17].

Migration clearly adds increasing complexities to the lives of IDPs and children. Hence, research that elucidates the plight of migrant children in troubled countries such as Ethiopia is urgently needed. Obtaining data from children and adult IDPs is relevant as it provides an in-depth understanding of the children’s vulnerabilities that can, in turn, inform migration policies and programming responses. Hence, this study examined the insecurities experienced by IDPs in the Burayu camp in Ethiopia, and how they navigate and challenge them.

## Theoretical framework

We use an intersectionality approach to examine the insecurities of internally displaced children in Ethiopia. Intersectionality emerged from the Black feminism movement to address ideological structures where discrimination against women based on gender and sex, race, ethnicity was framed. [18] Therefore, intersectionality interrogates social inequality, race, class, gender, ability, sexuality, and ethnicity as power systems, and not identity categories. [19] Intersectionality looks beyond the demands for inclusion and exclusion sameness and difference and posits that people can simultaneously experience advantages and disadvantages. [20] Hence, the intersectionality lens makes visible social inequalities such as age, gender, class, nationality, and discrimination that the migrant children in Ethiopian IDP camps experience at different times and spaces to determine the outcome on their livelihoods.

Community is not static; therefore, a more dynamic social process [21] that examines the vulnerabilities of IDPs that pose socioeconomic insecurities could provide insight into structures of power in which they are located. Diverse aspects that include social locations, identity, gender, age, poverty, lack of access to education, and political forces could simultaneously intersect in complex ways and at different levels to impact the everyday experiences of IDPs, determining their state of health and well-being. Therefore, we acknowledge these social aspects throughout the research, including the analysis of data. IDP children in Ethiopia experience physical, societal, and environmental stresses from displacement, rendering them vulnerable to physical, emotional, and mental health issues. Hence, employing an intersectionality perspective provides a comprehensive analysis of how the multiple disparities intersect to influence the migration experiences and individual lives of these migrant children.

## Methodology

We conducted an exploratory qualitative case study [22] research to explore the forms of insecurities experienced by IDP children in Ethiopia. Qualitative inquiry is primarily undertaken when there is a need for a complex detailed understanding of a problem within a given context [23].

A case study approach allowed us to create boundaries, [22] around the case of children who have been internally displaced in Ethiopia, living in Burayu camp, Ethiopia. For each case, we interviewed a child and parent, or guardian. In addition, we interviewed policy makers and service providers in Burayu town Ethiopia. The intersectionality framework provided a lens to recognize how power and power relations across intersecting social locations are reproduced and how this power relations may affect the IDP children in Ethiopia. The final written report includes the participants’ voices, reflections of the researcher, and interpretation of the problem [23].

The following research questions guided this study:

1. What are the forms of insecurities and vulnerabilities experienced by IDP children in Burayu camp, Ethiopia?
2. How do IDP children in the Burayu camp experience the effects of their insecurities?

### Ethics

We obtained ethics approval from both the University of Alberta, Canada and Jimma University, Ethiopia. Participants provided voluntary informed consent before participating in their interview. For children 12 to 17 years, informed consent was obtained from their parents or guardians and assent was obtained from the child. Participants were assured confidentiality throughout the study process. Each participant received 180 Birr (approximately USD$4) for lunch and 83 Birr (roughly USD$2) for their transportation costs.

### Sampling and participant recruitment

We used a purposeful sampling approach by selecting information-rich cases. [24] We hired a community worker to support participant recruitment. Recruitment started December 2020 to February 2021. The participant population was IDPs living in an IDP camp in the Oromia Special Zone called Burayu camp as well as service providers working in Burayu town. The service providers were healthcare professionals providing direct services to the IDPs and managers/coordinators at the Oromia regional health bureau. Burayu is a town on the outskirts of Addis Ababa, the capital city of Ethiopia. Table 1 presents characteristics of the participants. Children interviewed were between ages 12years and 15years, parents were between ages 25years and 75 years, and service providers were between ages 27 years and 46 years, 70% of the children lived with both parents, and 90% of the parents were married, 21% of the female parents had a high school diploma compared to 83% of the male parents, and 65% of parents reported no source of income.

**Table 1:** Participant Demographics

Participants	Age	Gender	Level of education	Marital status	Living status	Income status
Children	12–15 yrs	Female-12 Male-8	**Grade 2–3** Female-5 Male-5 **Grade 4–6** Female-7 Male-3	Married-0	Both parents-14 Mother-6 Father-0	No income-20
Parents	25–75 yrs	Female-14 Male-6	**Illiterate** Female-11 Male-1 **≤** **High school** Female-3 Male-5	Married-18 Divorced-1 Widowed-1	With spouse-14 Female only-6 Male only-0	No income-13 Some income-7
Service Providers	27–46 yrs	Female-4 Male-9	NA	NA	NA	NA

* yrs = years

### Data collection

Interviews were conducted by author MS and a research assistant who are bilingual English and Afan Oromo speakers. We conducted all interviews at a time and location chosen by the participants. We conducted interviews with 20 children (CP), 20 parents/guardians (PP), and 13 service providers (SP) using a semi-structured interview guide that explored individual and family experience of insecurities during and after migration. Interviews were conducted in Afan Oromo and were audio recorded and transcribed in Afan Oromo, then translated into English. Two interviews were back translated to assess the accuracy of the translation. Each interview session lasted between 60 and 90 min.

### Data analysis

We used NVivo 12 qualitative data analysis software to analyze the data following Braun & Clarke’s [25] thematic data analysis approach. Thematic analysis is a method for identifying, analyzing, and reporting repeated patterns of meaning (themes) across a dataset. Two authors (MS and JK) independently read three transcripts and developed a coding framework, which reviewed by author BS. Author JK read all transcripts and completed coding using the coding framework. Analysis of data was led by author J.K. Yin [22] recommends the use of theoretical proposition in the analysis of case studies. The theoretical proposition of this study was based on the intersectionality perspective to account for how diverse forms of insecurities were perceived and experienced by IDPs in Ethiopia to influence their migration experiences and lives. The unit of analysis for the case was the family unit.

## Results

The experiences of internal migration significantly contributed to the life experiences of the participating IDP children. The IDP children in Burayu town faced many challenges related to poor socio-economic conditions that exposed them to several insecurities related to access to health and healthcare, education, food, basic needs, and physical and emotional safety. This section presents the findings under themes that address the following socio-economic insecurities: (1) basic needs, (2) healthcare, (3) academic, (4) economic, (5) food, and (6) physical and mental health.

### Theme 1: basic needs insecurity

This theme describes challenges the participants face with respect to accessing basic needs, such as healthcare, shelter, clothing, sanitary facilities, and clean water. Access to basic needs is uncertain within the IDP population, diminishing future prospects and causing physiological and psychological health issues related to the inability to provide for themselves and their children. This section has two sub-themes: (1) clothing and shelter, (2) clean water and sanitary facilities.

#### Clothing and shelter

Due to their unplanned migration, participants often did not have a chance to secure their personal belongings; most fled their homes with only the clothing on them and could not obtain new clothing. Therefore, they are now dependent on humanitarian aid or the hosting communities for basic needs. The following participant explains:

We passed via different challenges such as lack of clothes and food. Primarily, we migrated without taking even a single cloth. However, after we traveled to Chiro, our community brought clothes for us. Similarly, the same thing happened when we came to Burayu. In addition to this, the government also supported us by bringing materials for food. But now the government stopped the support. (CP005)

Participants were also concerned with the poor living conditions at the IDP camps. The housing structures were made from materials that did not provide adequate shelter from the heat during the day and the cold at night. Further, migrating from a warmer to a colder region made it difficult for parents to protect their children from the cold when they did not have access to warm clothing and blankets. As this participant explains:

Genuinely speaking, I don’t like this town. This town is too cold; it is after people started clearing forests that the cold is improved. Due to the coldness of this area, we are exposed to various diseases. We were brought up in a hot climate. There, we do not even wear blankets during the night. We are adapted to this kind of climate. (CP020)

What is difficult is now is this shelter, it absorbs sunlight and become too hot during the daytime. And, during the nights, it becomes cold as ice. (PP018)

#### Clean water and sanitary facilities

Participants expressed a lack of adequate access to sanitary facilities and clean water. This led to poor hygiene and sanitation within the camps. The shelters did not have proper waste and drainage systems, and the latrines were built far from the shelters, making them hard to access at the night. The following participant explains:

Previously, our house was not like this, and we do not have a separate kitchen and toilet and we were defecating on the field. However, there are a lot of hyenas around here, so it was risky to go to the field during the nighttime. (CP008)

Participants also indicated lack of a sufficient and regular supply of drinking water, which increased the risk of waterborne diseases such a cholera:

Frankly speaking, I can say we are not living. Having nothing to eat and only seeing each other cannot be considered as life…lack of water. We are not getting enough water to drink. It comes once per week, sometimes even longer. They release it at midnight, and we have to wait for it to fetch. We also have no food. (CP015)

### Theme 2: healthcare insecurity

This sub-theme describes participant experiences accessing healthcare. The IDPs were initially provided free access to healthcare by the government through local health facilities. However, the support has been discontinued because the IDPs are expected to be independent and assimilate into the local community.

We opened a clinic in the camp to provide services until they become independent. The healthcare providers were assigned to the clinic and providing service there. If it is beyond the capacity of these health professionals, they refer to nearby health centers and hospitals. On the other hand, health extension workers were assigned to provide health education to these people. All services from health centers and hospitals are free of charge for these people. After these people became self-dependent all free health services were stopped. However, health education and follow-up at the camp are continued by health extension workers. (SP008)

Three sub-themes are discussed in this section: financial barriers, lack of drugs, and poor quality of care from the health facilities.

#### Financial barriers

Contrary to service provider information, participants indicated they did not have access to free healthcare services. The lack of access to healthcare prevented the proper treatment and management of health conditions. For example, the following participant indicated they preferred to stay home and manage their health conditions because they could not afford healthcare services:

I had got ill for two days. One day I was injured by steel, during fasting, and slept for the full month…I didn’t go to a health facility due to a lack of money. They don’t give service freely. My family just bought alcohol (GV) for me since we have no money to go to a health facility. (CP013)

Conversely, other participants stated they could access health services for free: “Regarding health services, they also provide me different care like medicine and plumpynet when I feel pain. Even if we do not have money, they buy the medication for us as we need” (CP008). But this is not the case in all health facilities, as explained by the following participant:

At the beginning, we had free services. Recently we do not go to a health center in Burayu because recently they started asking for money, there is no drug, they ask us to pay for a card and an ambulance cannot come if we call them. (PP008)

Participant responses about access to healthcare showed a disconnect between the available services for IDPs and their awareness of service availability. For example, one service provider indicated the following:

They are getting free services. They are getting free services from health facilities in our town after getting a supporting letter from our office. If they have to buy medication in our health facilities, our town municipality buys for them. If they can get it from the health center, they get it freely then based on the slip the government pays on behalf of them. (SP011)

Another service provider indicated the IDPs were now considered self-dependent and as such were expected to pay for healthcare services. This knowledge gap regarding access to or awareness of healthcare service provision caused mortalities among the IDP population. The following participant highlights the financial challenges IDPs faced related to healthcare access:

Recently my brother was bitten by a dog. We had no money to take him to the hospital and we just let him stay at home. Then his illness worsened and he passed away. If we were at Jigjiga (their home) we would have helped him since we had money. He was 5 years old. We could not help him. We have been in a difficult situation. (CP011)

As such, a sense of community cohesion to combat the financial barriers to children and IDPs accessing healthcare services was evident within this group. The following participant explains how the members of the community pooled together to collect money for them to receive treatment after sustaining an injury that required hospital treatment:

We have been allowed to be freely served at health centers. Yet, it was just for publicity; their service was very poor. Now, when we get sick, we take a loan from those who have money to cover the expenses. Our children also know the situation we are in; thus, they do not push us a lot. (PP014)

#### Lack of drugs

Some participants indicated they were supposed to have access to free drugs, but because the health facilities consistently lacked drugs they were referred to a private pharmacy to purchase medications even though they did not have money to buy them. The following participant expounds:

There is no drug at the health center. They (referring to health centers) prescribe medication to private pharmacies where we have to pay. We have no money, the health center says, “Your budget is already completed!” (PP003)

Even when the medicine was available at the pharmacy, participants denoted a lack of responsive care by healthcare providers, who treated them differently because they were IDPs. The following participant explicates:

As I said earlier, when we came to this place, we were new to the environment and we faced many challenges and exposed to different diseases. So, we visited health institutions many times. One day my young child get sick and I took him to a health center, however when I reach there the health care provider (pharmacist) told me that, there is no medication because there are no medications for displaced people. I came back home with syrup and tablet, but my child did not get improvement by that syrup. Then I went to the health institution again after two days. The health care provider (Pharmacist) complains again about me by saying “you came here so many times. You already finished your medications, so I don’t give you any medication.” He also insults me by saying “you were displaced because of your behavior”. (PP007)

#### Poor quality of care

A lack of trust and respectful relationships with healthcare providers was evident among participants, indicating a need for people-centered care and community engagement to ensure patient safety and optimal treatment. Some participants noted the poor quality of services at the health facilities, poor attitudes of staff towards IDPs, and inconsistent delivery of healthcare services by providers. Participants articulated discrimination from some healthcare staff and found the process of seeking healthcare from some health facilities daunting. This prevented participants from accessing healthcare care services, with them instead reverting to traditional medicine:

When we go to the health center, they insult us. So, we simply use traditional medicine. Most of the time if it is not a must, we do not go to health centers in [XXX] rather we go to [XXX]. Health professionals who are working in [XXX] health center become angry and their face is not welcoming, and it is difficult communicate with them. Also, they do not make eye contact with us, whenever they hear we are displaced communities. (PP008)

Similarly, participants indicated they did not receive appropriate assessments and treatments at the health facilities, which could exacerbate their illnesses. Others were concerned about receiving inappropriate drugs because they received the same kind of drugs for different conditions. The following participant explains:

Yes, since no fee they give us the same type of drug for different diseases… no problem but they give the same drug for both adult and children… they give us penicillin for all diseases. (PP010)

In some cases, unresponsive care contributed to maternal and infant morbidity and mortality. For example, one participant reported an infant death occurred due to the unavailability of transportation to the health facility: “last week we tried to call an ambulance for a pregnant mother going to give birth and they didn’t come. She delivered at home and unfortunately, the baby died” (PP008).

### Theme 3: academic insecurity

IDP children who were unable to enjoy basic education and normal growth opportunities because they were living in an IDP camp captured the disadvantages that war and displacement bring to both accompanied and unaccompanied minors. Children lacked continuous schooling, interrupting their education trajectories. One of the reasons commonly indicated by the participants for not attending school was discrimination by the host community:

Our children were not allowed to attend school. If they registered to attend, they are exposed to discrimination and torture. We kept our children at home without schooling. Language in school is Somali language only. (PP002)

Cultural and linguistic barriers influenced IDP children’s access to education. Children felt intimidated and fearful of attending school with children from the host communities because of perceived hostility. The following participant explains how the IDP children separated themselves from the local students at school during mealtimes:

In school, during break time, I stay with students displaced. Because displaced students do not have money to buy things they need like local students. When local students play and eat together, we try to separate ourselves from them because we can’t buy a single thing. At that moment either we stay in the classroom or go far from their places not to look at their hands. (CP005)

We also noted that children were enrolled in lower primary classes regardless of being older than this level of education. For example, despite all child participants being at least 12 years of age, the following participant was in grade 3:

He was in grade 2 when he was in Jigjiga [now in grade 3]. He missed 1 year when we were displaced. After coming here, the government gave them a school uniform and they were enrolled. All of a sudden, it was again interrupted because of COVID-19. (PP001)

Other children stopped attending school due to a lack of essential school supplies such as uniforms, as well as interruptions caused by migration. Those who attended school were concerned about security challenges in the area interfering with their concentration in school:

What is challenging for him is the violence in this country. Children are very much concerned about security issues. Education needs concentration. On top of this, the scarcity he sees always in the house greatly impacts his performances. Otherwise, he tries to balance between his leisure and studying time. His concerns are what to wear and eat. (PP001)

The following participant, who is a public servant, explained how they provided support letters to local schools to accommodate IDP children. Most of these children dropped out of the school regardless of the free education and support provided, such as free supplies and uniforms:

Some students did not come after they registered and entered the system. We were facing this problem at different times. Additionally, more than those who did not come there are many drops out. They come today and absent tomorrow. They come today and absent the next day. There is such experience. It is known that this can bring a potential impact on the futurity of these children. (SP013)

Disruption of education could have long-lasting consequences for these children and jeopardize future opportunities. The loss of property and sources of income encountered by families because of displacement could also contribute to children seeking work instead of going to school. Hence, children may discontinue school due to lack of food and inadequate support from home:

They discontinued their education. Also, the support they are getting is not adequate, so they may not live as at home. They may directly face problems. The problems could be lack of food and the support they are getting is not enough. Also, these children may engage in work beyond their age. This work can affect their education and health. (SP001)

### Theme 4: economic insecurity

Loss of income rendered the parents helpless with respect to providing their children with basic necessities. The participants indicated the loss of income due to the inability to find jobs within their new communities made it difficult to provide for themselves and their children. As men are typically the breadwinners within families, some moved to other cities searching for jobs, leaving women and children behind at the IDP camps. Leaving women to act as the heads of households consequently heightened the vulnerability of both women and children due to the altered family roles. The separation could also contribute to stresses within the family structure, as explained by the following participant:

We are facing a lot of problems. We are not much familiar with the environment to work. This area is a bit different. Finding a job is difficult. The money my husband is sending us is not enough. My children ask me for many things. And I can’t afford what they want. (PP012)

The lack of necessities due to loss of income put pressure on both children and their parents. Most participants expressed they would do all they could to provide for their children. Unfortunately for the children, when their parents found jobs, the wages were too meager to meet their needs and the children were forced to take over home activities such as cooking, as the following participant indicates:

It is difficult to live with eight children alone. But I decided to work whatever I get to get some money. You know it is hard to afford soap for these all children. I tried all my best to survive… My children would have been on the street if I did not work hard. I think about my children’s mindset. I worry about them feel helpless. Therefore, I try hard to give them a few from what they should get. My child is very kind and he helps me a lot. We come back home from work, I get almost everything prepared for dinner. He prepares it. (PP018)

Further, some participants indicated they were more in debt because they had diverted the loans the government had granted them during the COVID-19 pandemic, placing them in default. The following participant preferred to be back in their home regardless of the violence:

The government has organized us into a small group and gave us money to start work. But before we start work, Covid-19 started. Because of the nature of the diseases, we stayed home. When you stay home without a job, spending money without reason is common. Now, almost we finished the money we got from the government until now we did not start any work. So, for how long I will stay here without a job? This situation stressed me a lot. I do not compare what I got here with what I had while in Wuchale. I never get what I was getting in Wuchale even if I work my entire life here. Therefore, I want to return to Wuchale because rather than dying by lack of food here, I prefer to die on their hand (referring to Somali) with a satisfied and nourished body. (PP016)

### Theme 5: food insecurity

This theme speaks to the lack of consistent food supply to participants living in IDP camps. The lack of constant food supply was a general problem affecting IDP children. Depending on humanitarian aid, participants stated they often did not have enough food to eat and water to drink. Moreover, not everyone could access humanitarian support, leaving children and the IDP population hungry. The lack of food threatens children with malnutrition and starvation, increasing the risk of morbidity and mortality among the IDPs, especially children and breastfeeding mothers.

The following participant explains how lack of consistent food supply affected their daily routine and threatened their survival at the IDP camp. This participant felt that, despite other problems the IDPs faced, lack of food was their primary concern:

Yes, we faced hunger. We don’t have any other problems except lack of food. Even right now, I didn’t have my breakfast, we have nothing in our house. We don’t have money to buy food and feed ourselves. So today no breakfast. I don’t know how I cope with that. (CP002)

The lack of constant food supply caused IDPs to resort to unhealthy coping practices such as skipping meals. This practice is harmful to children because they need sufficient nutrition for growth and development. The following participant explained: “Currently, I have five children. If I get money, I buy something and feed them, if not we go to sleep without food. We are at high risk of hunger and cold” (PP005). Children’s physical and daily activities were also inhibited by hunger and lack of a balanced diet, contributing to poor body functioning and growth. For example, this participant explains how some children could not participate in play or learning because they did not have sufficient food to eat: “I have nothing to do other than simply sitting. I didn’t play. How could I play with hunger? With an empty stomach how can I play?” (CP013).

Participants also noted the lack of access to basic needs and financial barriers to meet these needs contributed to their decision to trade the food received from humanitarian food aid. The lack of other necessities such as hygiene products and clothes as well as medical expenses forced them to sell part of the food ratio they received, placing children at even greater risk of malnutrition.

They have been providing us ration when we came here…I have five children; we are 6 together with me… I have to sell from this monthly ration to get money and buy other necessities like soap, fruits, clothes, shoes and the like. I also use it for medical expenses…We can do nothing with the rice they give us. It needs at least salt to be eaten. (PP013)

### Theme 6: physical and mental insecurity

This theme concerns the physical violence the participants either experienced or witnessed, which impacted both their physical and mental well-being. During the migration process, most participants sustained physical injuries, witnessed killings of other migrants, and faced abduction from their homes. The following participant explains how physical insecurity disrupted economic activities and inflicted trauma on the IDPs:

One day I was looking after the Cattles. Since the conflict between Oromos and Somalis was on spot, I was hearing a gunshot. I was very afraid. The war comes closer to where I was and I witnessed one of the cows shot and died in front of me. I fainted there and I did not know who took me to our home. Additionally, Somali children brutally kicked me and my friends by bending us in a line. It was very difficult. (CP018)

The participants narrated the violence they witnessed prior to migrating to the camp, which led to their decision to relocate. They felt intimidated by and helpless in the face of the brutal killings of people including women and infants. Such exposure to violence can affect children’s mental health in the long run. The violence also created uncertainty within the family unit, and children were fearful as some were wounded during migration. For example, the following participant described their experience:

Yes, I have seen it, and they (Somali) killed him (Oromo man) while he was coming to our house, at that time we sat in our compound, and when we heard the sound of people and run to him, he was dead… I have seen while they killed one woman using a knife and gun, and fortunately, her child was saved by another person. (CP007)

The fear initiated by previous violent experiences interfered with the children’s social interaction at the camps, affecting their quality of life and normal growth. For instance, this child participant expressed fear of being harmed if they played with other children in the camp: “I felt that children may harm me when I play with them on the field. Due to this, I don’t want to enter into conflict with children while we are playing” (CP007).

One disturbing issue was the concerns women and girls had about being victims of rape. One of the healthcare participants indicated that raped women or girls could not disclose their experiences due to fears of losing their families. They described this as “a problem hidden, and you see the tip of the iceberg” (SP012). Rape and the stigma from rape exposes girls and women to potential untreated sexually transmitted infections such as HIV and could also cause long-term reproductive and psychological effects.

Raped females do not tell you because they do not want to lose their family. Their husband could hear about the situation, but her husband may die, so rather than telling the history, they prefer to hide but hate males. After a long time, if they do not get a person who they can tell their history, they may kill themselves. (SP012)

Our data analysis shows that socioeconomic and contextual factors intersect to determine the health and well-being of children in the Ethiopian IDP camp studied. The children experienced insecurities while navigating their daily lives. This is compounded by institutional practices that shape gender relations, income status, and access to healthcare, education, and food.

## Discussion

This paper exposes the basic, academic, economic, and physical and mental insecurities and vulnerabilities experienced by children living in an IDP camp in Ethiopia. Similar to other studies, ours shows the IDPs face many precarious and distinctive health challenges, such as limited resources, including food, water, shelter, and security [7], [26], [27], [28]. Institutional practices that shape gender relations, income status, access to healthcare, access to education, and access to food compound the experiences of IDPs, leaving their basic needs unmet. For instance, for some IDPs, access to healthcare services was impeded by lack of finances, unavailability of drugs, and poor-quality service at healthcare facilities. Some IDPs were able to access free healthcare while others could not, indicating IDPs can experience these insecurities differently. Similarly, the disruption of essential services such as banking, fuel, and logistical challenges can cause disruptions in shipments of critical supplies (e.g., drugs) to IDPs in Ethiopia [29].

Further, income insecurity has negatively aggravated access to basic needs such as food, shelter, clothing, and healthcare, causing uncertainty in IDP livelihoods. Other studies indicate that lack of access to basic needs diminishes hope and causes psychological health issues for parents and children, [30], [31] as related to the inability to provide for themselves. Therefore, there is a need to ensure IDPs are aware of the social and healthcare services available to them.

Internally displaced children are at risk of malnutrition, [11], [32] which can lead to lifelong adverse effects. The participants in our study acknowledged receiving food relief, but the inconsistencies in food supply contributed to frequent food shortages. These food shortages led to skipping meals to preserve the little food to which they had access. Our study also noted that loss of income led to IDPs engaging in other creative ways to obtain money, such as selling part of the food received from relief agencies to buy other necessities, further aggravating food shortages. Hence, due to unhealthy dietary behaviors related to food insecurity, immigrant children are prone to physiological problems such as anemia, vitamin D deficiency, wasting, and stunting, among others. [9] Grijalva-Eternod et al. [33] note that policies and interventions such as cash transfers could prevent child malnutrition and improve overall household health.

Children deserve opportunities that promote regular growth and learning, including access to affordable education. Unfortunately, the ability of IDP children to obtain an education was impeded by a lack of basic school supplies, language barriers, and constant movement. Most children in our study were enrolled in lower primary classes regardless of age and previous education level. Hence, constant movement and language barriers deny IDP children a good school foundation, leading to limited opportunities for a prosperous livelihood, including knowledge of health practices and financial security. Education is also a good platform for empowering the decision-making ability of women and girls. [34] Given that internal displacement is usually long term, it is essential to ensure children in these situations continue to learn. For example, in Syria, parents were equipped with skills to help the children learn when unable to attend school [35].

Loss of parents or poor parental care requires sustained attention to realign available resources with children’s psychosocial needs during post-conflict periods. Early economic reintegration could benefit IDPs and potentially prevent the severity of the social and health disparities they face due to migration. Due to separation-related income generation efforts, social integration problems are common in most IDP family situations. [36], [37] Our study shows some IDPs initially received cash loans from the government to help with resettlement. However, due to lack of income, most diverted the loans during the COVID-19 pandemic period, placing them in a default situation. As such, husbands/fathers were forced to move to other cities to look for employment, leaving women to head the households. This points to the need to manage and follow up on economic reintegration efforts within the short and long-term using an intersectionality lens. These efforts must integrate gender relations and include activities that will help achieve goals for specific projects attached to donor support outcomes, consequently enabling social support and conflict resolution management efforts. For instance, some organizations are now tasking women with the appropriation of humanitarian assistance received in the IDP camps, including food aid. [37] Women have proven to better understand and prioritize the needs and realities of different groups within the recipients of humanitarian aid, such as women and girls [38].

Safety was also a significant issue for children and IDPs, causing disruption of economic activities and children’s developmental progress, including education. Further, violence and physical insecurities have created homelessness among the IDPs, eliminating their sense of belonging within the country context. IDPs can also face armed attacks and sexual abuse within the IDP camps. Participants explained how societal norms exposed women and girls to health risks post-rape due to the stigma associated with sexual and gender-based violence in the community. Studies from African contexts have raised concerns about these risks for women and girls. For instance, one qualitative study that explored the cartography of safety and risk within community spaces among parent/caregivers and adolescent girls in South Kivu [39] found elevated fear of sexual and other forms of gender-based violence within the constrained environment in which the IDPs live. Their study also found that boys and men dominated the public spaces, reducing the girl’s structural support to challenge the domination of male areas. Sexual perpetration is not only conducted by men and boys living in the IDP camp but also by humanitarian staff, as documented in Sierra Leone, Liberia, and Guinea [40].

As such, it is imperative to educate humanitarian staff and the community about the criminality of gender-based sexual abuse and violence and the severity of laws and punishment for those committing such acts. Women and children are exposed to mental health issues related to fears of gender-based violence. In most societies in Africa and other communities, a victim of rape is considered immoral. [39] Thus, there is a need to build safe spaces for girls and women caregivers living in IDP camps and, more importantly, to empower the girls and caregivers and educate the community on the need to change the negative attitudes and behaviors that affect girls and women.

## Study strengths and limitations

Using a semi-structured interview guide allowed for participants to provide detailed and rich data regarding the study phenomenon. A strength of our project is that we engaged in data source triangulation by interviewing both parents and children. Yin [22] suggest using multiple data collection sources. However, our unit of analysis for the case was the family, and participants provided rich interview data. We did not triangulate methods. We have included that future studies should consider methodological triangulation. We also acknowledge that the documented responses and experiences may not be generalized to all IDP camp settings, however, findings can be transferrable to other settings.

## Conclusion

This paper describes how Ethiopian children living in Burayu town IDP camp experience vulnerabilities and navigate their daily lives. Multi-level strategies are needed to reduce the socioeconomic insecurities and vulnerabilities among the IDP population. These strategies include the need to continue supporting the IDPs by ensuring seamless logistical support (e.g., supplies) from governmental and non-governmental organizations throughout the migration process. Most importantly, there is a need to build confidence amongst the IDPs in governmental efforts to provide temporary comfort within the IDP environment. Engaging an intersectional lens when developing policies and processes will reduce bias and discrimination against specific populations such as IDPs. With transparent processes, IDPs would be well informed about social and humanitarian supports and access to these services. Successful relocation and reintegration of IDPs would help limit both parent and child post-conflict trauma.

## Data Availability

The datasets generated during and/or analysed during the current study are available from the corresponding author on reasonable request.

## Abbreviations

CP: Child Participant
IDMC: International Displacement Monitoring Center
IDP: Internally Displaced Persons
PP: Parent Participant
PTSD: Post-traumatic Stress Disorder
SDG: Sustainable Development Goals
SP: Service Provider
UNHCR: United Nations High Commission on Refugees
UNOCHA: United Nations Office for the Coordination of Humanitarian Affairs
